# Purified Compounds from *Phyllanthus amarus* Schum. & Thonn. Inhibit Mast Cell Degranulation via Modulation of Syk/PLCγ Signaling

**DOI:** 10.3390/molecules31162835

**Published:** 2026-08-14

**Authors:** Thanh Sang Vo, Vo Thi Ngoc My, Phan Van Hoai Luan, Dai-Hung Ngo

**Affiliations:** 1 Center for Hi-Tech Development, Nguyen Tat Thanh University, Saigon Hi-Tech Park, Ho Chi Minh City 700000, Vietnam; vtsang@ntt.edu.vn (T.S.V.);; 2 Interdisciplinary Science Institute, Nguyen Tat Thanh University, Ho Chi Minh City 700000, Vietnam; 3 Faculty of Medical Laboratory, Nguyen Tat Thanh University, Ho Chi Minh City 700000, Vietnam; 4 Department of Chemistry, Thu Dau Mot University, Ho Chi Minh City 750000, Vietnam

**Keywords:** anti-allergy, degranulation, histamine, mast cells, *Phyllanthus amarus*, Syk

## Abstract

*Phyllanthus amarus* has long been used in traditional medicine and contains diverse bioactive compounds with potential pharmacological properties. This study aimed to purify the compounds from *P. amarus* and investigate their inhibitory effects on mast cell degranulation in vitro. It was found that the ethyl acetate fraction from *P. amarus* possesses potential inhibitory effects on mast cell degranulation. Subsequently, three compounds, including 3,3′,4-tri-O-methylellagic acid (P**1**), ethyl brevifolincarboxylate (P**2**), and 4′,4′′′-di-O-methyl cupressuflavone (P**3**), were purified and identified. These compounds significantly inhibited mast cell degranulation via decreasing histamine release and IL-4 and TNF-α production from the activated mast cells without any cytotoxicity. Furthermore, Western blot analysis confirmed that these compounds suppressed phosphorylation of Syk and PLCγ, suggesting inhibition of the degranulation-related signaling cascade. These findings provide preliminary in vitro evidence that purified constituents from *P. amarus* may inhibit mast cell degranulation, potentially in association with modulation of Syk/PLCγ signaling, and support further mechanistic and in vivo investigations.

## 1. Introduction

*Phyllanthus amarus* Schumach. & Thonn. is a small annual herb belonging to the family Phyllanthaceae, widely distributed throughout tropical and subtropical regions such as India, China, Brazil, and Africa. It typically grows up to 60 cm tall, with slender stems and numerous small green leaves arranged alternately along its branches (Patel et al. 2011) [1]. This plant has been traditionally used in various ethnomedical systems, including Ayurveda, Unani and traditional Chinese medicine [2]. It is traditionally prescribed for the treatment of liver disorders such as hepatitis B and jaundice, kidney and gallbladder stones, malaria, diabetes, dysentery, urinary tract infections, respiratory problems, and skin diseases [1]. Phytochemical investigations have revealed that *P. amarus* contains a wide spectrum of secondary metabolites that contribute to its medicinal properties. Lignans are the signature constituents, especially phyllanthin, hypophyllanthin, niranthin and hinokinin, which are considered key chemomarkers of the genus. The plant also contains abundant polyphenols and tannins, notably geraniin, corilagin, ellagic acid, and gallic acid, which possess strong antioxidant capacity. Flavonoids such as rutin, quercetin, and kaempferol have also been detected and are known to have anti-inflammatory and free radical scavenging properties. Additionally, several alkaloids including securinine and epibubbialine, together with triterpenoids such as lupeol, ursolic acid, and sterols, have been reported [1,3]. Notably, *P. amarus* exhibits potent anti-inflammatory properties through the inhibition of pro-inflammatory mediators including TNF-α, IL-1β, and IL-6 [4]. Recently, the antihistamine potential of *P. amarus* was assessed using a competitive radioligand binding assay targeting the histamine H1 receptor (H1R). Analysis by high-performance liquid chromatography revealed four major active constituents, namely phyllanthin, hypophyllanthin, niranthin, and corilagin, which exhibited potential antihistamine activity [5]. Despite increasing evidence supporting the pharmacological activities of *P. amarus*, the specific anti-allergic constituents and their underlying molecular mechanisms remain largely uncharacterized. In particular, the purification of active compounds and mechanistic validation in mast cell-based allergic models are still lacking. This gap limits the translation of *P. amarus* from traditional use to mechanism-based therapeutic development. Based on the reported bioactivity of polyphenol and lignan-derived compounds in *P. amarus*, we hypothesized that specific purified constituents from this plant exert anti-allergic effects by suppressing mast cell activation through inhibition of the Syk/PLCγ signaling pathway, a central cascade in IgE-mediated allergic responses. Therefore, the present study aimed to purify the compounds from *P. amarus* and investigate their inhibitory effects on mast cell degranulation in vitro.

## 2. Results and Discussion

### 2.1. Screening of P. amarus Fractions for Mast Cell Degranulation Inhibition

**Figure 1:**
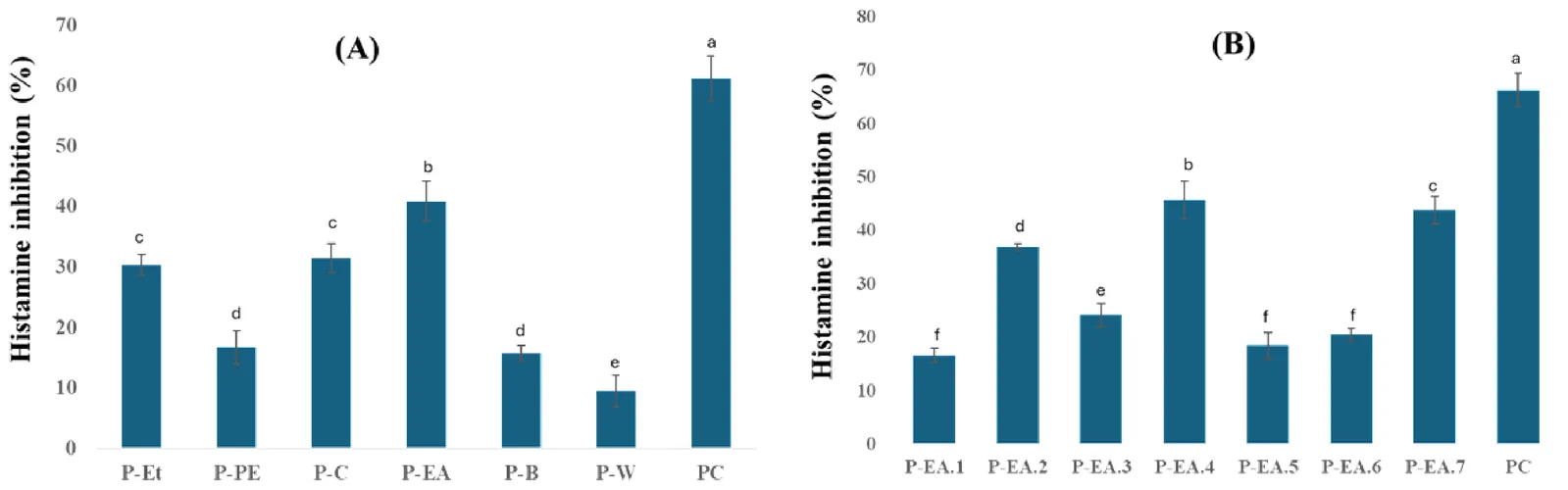
The inhibitory effects of *P. amarus* fractions (**A**) and ethyl acetate sub-fractions (**B**) on histamine release from RBL-2H3 cells. The cells were pre-treated with 100 µg/mL of samples and sensitized with DNP-specific IgE antibody before stimulation of antigen DNP-BSA. Histamine released from cells was measured using a fluorescence spectrophotometric assay. Each determination was made in three independent experiments, and the data are shown as means ± SD. Different letters a–f indicate significant differences among groups (*p* < 0.05) by Tukey’s multiple-range test. P-Et: 70° ethanol fraction; P-PE: petroleum fraction; P-C: chloroform fraction; P-EA: ethyl acetate fraction; P-B: butanol fraction; P-W: distilled water fraction. Cromolyn sodium (50 µg/mL) was used as positive control (PC).

In this study, a range of solvents with differing polarities, including petroleum, chloroform, ethyl acetate, butanol, and distilled water, was utilized to selectively partition compounds according to their polarity. This approach serves as a preliminary step for further mechanistic investigations and identification of bioactive constituents. The inhibitory effects of different solvent fractions of *P. amarus* on histamine release from mast cells at a concentration of 100 µg/mL are presented in Figure 1A. Among the tested fractions, the ethyl acetate fraction (P-EA) exhibited the most pronounced activity, achieving 40.9 ± 3.3% inhibition, which was significantly higher than that of the other fractions. The superior activity of the P-EA fraction may be attributed to the enrichment of medium-polarity polyphenolic compounds, particularly ellagitannins and flavonoids, which have previously been reported to exert anti-allergic and mast cell-stabilizing effects [6,7]. Ethyl acetate is known to selectively extract phenolics and certain glycosides, suggesting that these bioactive molecules may play a key role in modulating histamine release. Accordingly, the P-EA fraction was selected for further sub-fractionation.

The inhibitory activity of the sub-fractions derived from the P-EA fraction at a concentration of 100 µg/mL on histamine release is shown in Figure 1B. Among these, sub-fraction P-EA.4 exhibited the strongest inhibitory effect on histamine release from mast cells, reaching 45.7 ± 3.5% (*p* < 0.05). This was followed by P-EA.7 and P-EA.2, which also showed marked activities of 43.8 ± 2.6% and 36.9 ± 0.7%, respectively. Sub-fraction P-EA3 displayed moderate inhibition, whereas P-EA1, P-EA5, and P-EA6 demonstrated relatively weak effects. However, their effects remained lower than that of cromolyn sodium, which showed approximately 66% inhibition at a treatment of 50 µg/mL. The results indicate that the bioactive constituents responsible for the degranulation inhibition of *P. amarus* may be distributed within sub-fractions of P-EA.2, P-EA.4, and P-EA.7. The further purification of P-EA.2, P-EA.4, and P-EA.7 was therefore warranted to identify the bioactive components.

### 2.2. Purification and Identification of the Bioactive Compounds from P. amarus

**Figure 2:**
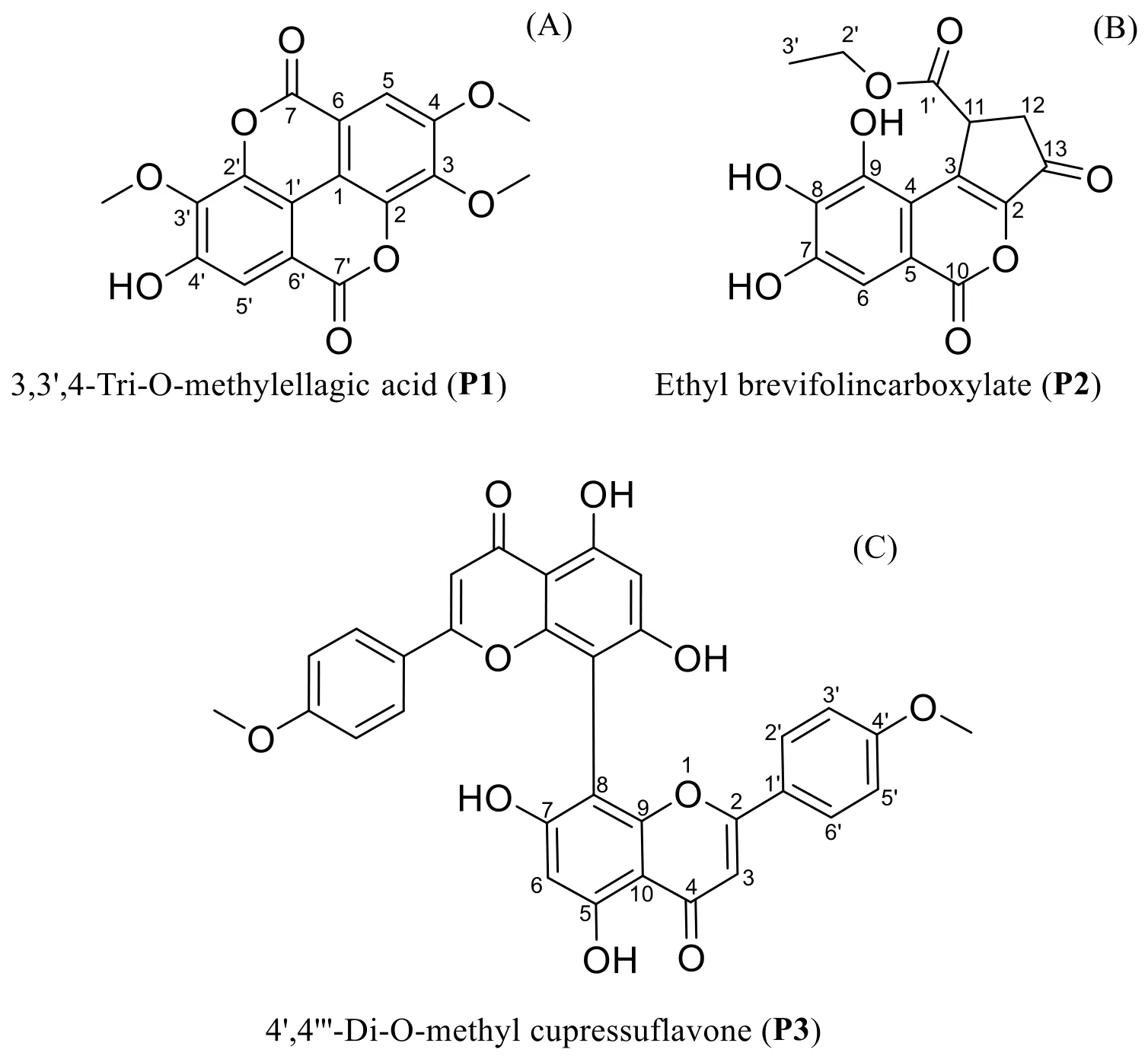
The chemical structures of P**1**, P**2**, and P**3**.

The compound P**1** was obtained as a pale-yellow powder. The ^1^H NMR spectrum of P**1** displayed only two types of signals: two aromatic protons at δ_H_ 7.51 (s, 1H) and δ_H_ 7.61 (s, 1H), together with three methoxy groups at δ_H_ 4.05 (s, 3H), δ_H_ 4.04 (s, 3H), and δ_H_ 4.00 (s, 3H). These data suggest that P**1** is an aromatic compound bearing three methoxy substituents. The ^13^C NMR spectra revealed the presence of 17 carbons, including two carbonyl carbons (δ_C_ 158.4 ppm and 158.2 ppm), twelve aromatic carbons (δ_C_ 111.1–152.5 ppm), and three methoxy carbons (δ_C_ 60.9, 61.2, and 56.6 ppm). Among the aromatic carbons, two were CH carbons (δ_C_ 111.6 and 107.4 ppm), four were quaternary non-oxygenated carbons (δ_C_ 111.1–113.3 ppm), and six were oxygenated quaternary carbons (δ_C_ 140.1–153.7 ppm). These features indicate the presence of two benzene rings. With two ester functionalities and two aromatic rings already established, the degree of unsaturation required by the molecular formula implies the existence of two additional rings. Collectively, these characteristics suggested an ellagic acid framework, and the compound was tentatively identified as a tri-O-methyl derivative of ellagic acid (Table 1, Figure 2A and Figure S1). Furthermore, the spectral data of P**1** were matched with those reported for 3,3′,4-tri-O-methylellagic acid by Gao and colleagues [8]. Thus, compound P**1** was conclusively identified as 3,3′,4-tri-O-methylellagic acid.

The compound P**2** was obtained as a pale-yellow powder. The ^1^H NMR spectrum of compound P**2** showed that a single aromatic proton signal was observed at δ_H_ 7.30 (s, 1H), corresponding to a benzene ring bearing five substituents. Additional high-field sp3 proton resonances included a methylene and methyl group of an ethoxy moiety at δ_H_ 4.09 (q, J = 7.1 Hz, 2H) and 1.17 (t, J = 7.1 Hz, 3H), respectively, as well as three other methine and methylene protons at δ_H_ 4.40 (dd, J = 7.7, 2.0 Hz, 1H), 2.98 (dd, J = 18.6, 7.7 Hz, 1H), and 2.44 (dd, J = 18.6, 2.0 Hz, 1H). The ^13^C NMR spectrum (DMSO-*d*_6_, 150 MHz) displayed 16 carbon signals. These included three carbonyl carbons corresponding to a five-membered ring ketone (δ_C_ 192.9), a lactone ester (δ_C_ 160.1), and an ethyl ester (δ_C_ 171.9), along with 13 aromatic and olefinic carbons bearing three hydroxyl substituents. These spectral characteristics suggested the structure of ethyl brevifolincarboxylate (Table 2, Figure 2B and Figure S2). Comparison of the obtained ^1^H and ^13^C NMR data with those reported in the literature further confirmed that compound P**2** was identified as ethyl brevifolincarboxylate [9].

The compound P**3** was obtained as a pale-yellow powder. The ^1^H NMR spectrum of compound P**3** exhibited aromatic proton signals at δ_H_ 7.11 (d, J = 9.0 Hz, 2H) and δ_H_ 8.04 (d, J = 8.9 Hz, 2H), corresponding to a symmetrically substituted benzene ring with para substituents. Additional signals included a singlet aromatic proton at δ_H_ 6.52 (s, 1H) and an olefinic proton at δ_H_ 6.88 (s, 1H). The ^13^C NMR spectrum (DMSO-*d*_6_, 150 MHz) revealed 14 distinct resonances corresponding to 16 carbons, consistent with a flavone skeleton bearing a symmetric benzene ring. A methoxy group was also evident at δ_C_ 55.5. Further correlations confirmed the core framework; notably, the presence of a C–C quaternary carbon signal at C-8 suggested a biflavonoid structure (Table 3, Figure 2C and Figure S3). Comparison of the ^1^H and ^13^C NMR data with the literature values confirmed the identity of compound P**3** as 4′,4′′′-di-O-methyl cupressuflavone [10].

### 2.3. The Inhibition of the Purified Compounds on Mast Cell Degranulation

**Figure 3:**
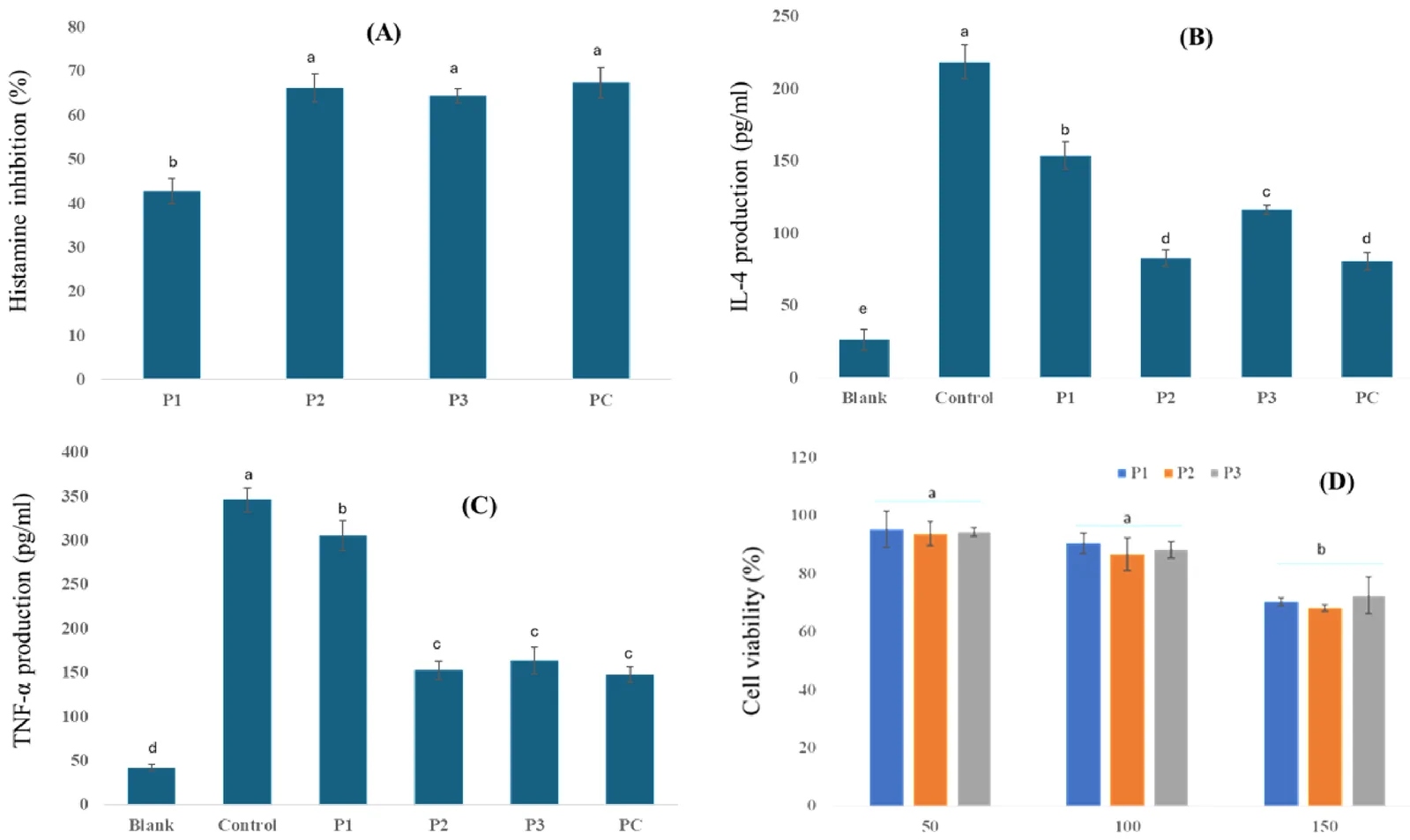
The inhibitory effect of the compounds from *P. amarus* on mast cell degranulation. RBL-2H3 cells were pre-treated with 100 µM of compounds and sensitized with DNP-specific IgE antibody before stimulation of antigen DNP-BSA. Histamine released from cells was measured using a fluorescence spectrophotometric assay (**A**). The production levels of IL-4 (**B**) and TNF-α (**C**) were quantified in culture media using commercial ELISA kits. Cell viability was examined by MTT assay (**D**). The blank group consisted of unstimulated cells, whereas the control group consisted of DNP-BSA-stimulated cells without compound treatment. Cromolyn sodium (100 µM) was used as the positive control (PC). Each determination was performed in three independent experiments, and the data are presented as means ± SD. Statistical significance was analyzed using one-way ANOVA followed by Tukey’s multiple-range test. Different letters indicate significant differences among groups (*p* < 0.05).

Inhibition of mast cell degranulation represents a key strategy for alleviating allergic inflammation [11]. In the present study, the inhibitory activities of the purified compounds from *P. amarus* on histamine and cytokine release from mast cells were evaluated at a concentration of 100 µM (Figure 3). It was found that all three compounds significantly suppressed histamine release (Figure 3A), with P**2** and P**3** exhibiting the highest inhibitory activity (66.2 ± 3.2% and 64.5 ± 1.6%, respectively), which were markedly greater than that of P**1** (*p* < 0.05). Cromolyn sodium, used as the positive control, showed 67.4 ± 3.5% inhibition of histamine release, which was comparable to the inhibitory effects of P**2** and P**3**. In terms of cytokine production, both IL-4 and TNF-α levels were markedly reduced in the presence of the tested compounds compared with the control (Figure 3B,C). Notably, P**2** exerted the strongest inhibitory effect, decreasing IL-4 production to 82.8 ± 5.8 pg/mL and TNF-α to 152.6 ± 10.3 pg/mL, while the control groups were 218.5 ± 11.6 pg/mL for IL-4 and 346.3 ± 13.8 pg/mL for TNF-α (*p* < 0.05), suggesting a potent role in downregulating Th2-associated allergic responses. The compound P**3** also demonstrated comparable inhibition, whereas P**1** showed only moderate suppression. Meanwhile, cromolyn sodium reduced IL-4 and TNF-α levels to 80.5 ± 6.1 pg/mL and 147.6 ± 8.8 pg/mL, respectively. These values were comparable to those obtained with P**2**, indicating that P**2** exhibited the strongest activity among the isolated compounds and approached the inhibitory effect of the positive control. Importantly, the observed inhibitory activities could not be attributed to cytotoxicity, as cell viability remained above 90% at concentrations up to 100 µM for all compounds (Figure 3D). This indicates that the inhibitory effects were specific to mediator regulation rather than non-specific cell death. The compound P**2**, a lignan glycoside derivative, possesses a polysubstituted benzene ring (bearing multiple –OH groups) and exhibits moderate polarity due to the presence of ester and phenolic groups. Meanwhile, P**3** is a biflavonoid, bearing two methoxy groups and multiple phenolic/hydroxyl substituents on the flavonoid backbone with moderate polarity. Numerous studies have determined the anti-inflammatory activity of lignans by suppressing NF-κB and MAPK pathways, reducing the expression of pro-inflammatory cytokines (IL-1β, IL-6, TNF-α) and enzymes such as COX-2 and iNOS, and enhancing antioxidant defenses [12]. In addition, flavonoids have been shown to exert potent anti-allergic activity by targeting mast cells, the key effector cells in allergic inflammation. They inhibit histamine release as well as the synthesis of Th2 cytokines such as IL-4 and IL-13, and suppress CD40 ligand expression [13]. Collectively, the compounds P**2** and P**3** from *P. amarus* may be suggested as promising natural agents for the modulation of allergic and inflammatory responses.

### 2.4. The Suppressive Effect of the Purified Compounds on Mast Cell Degranulation-Related Signaling Molecules

**Figure 4:**
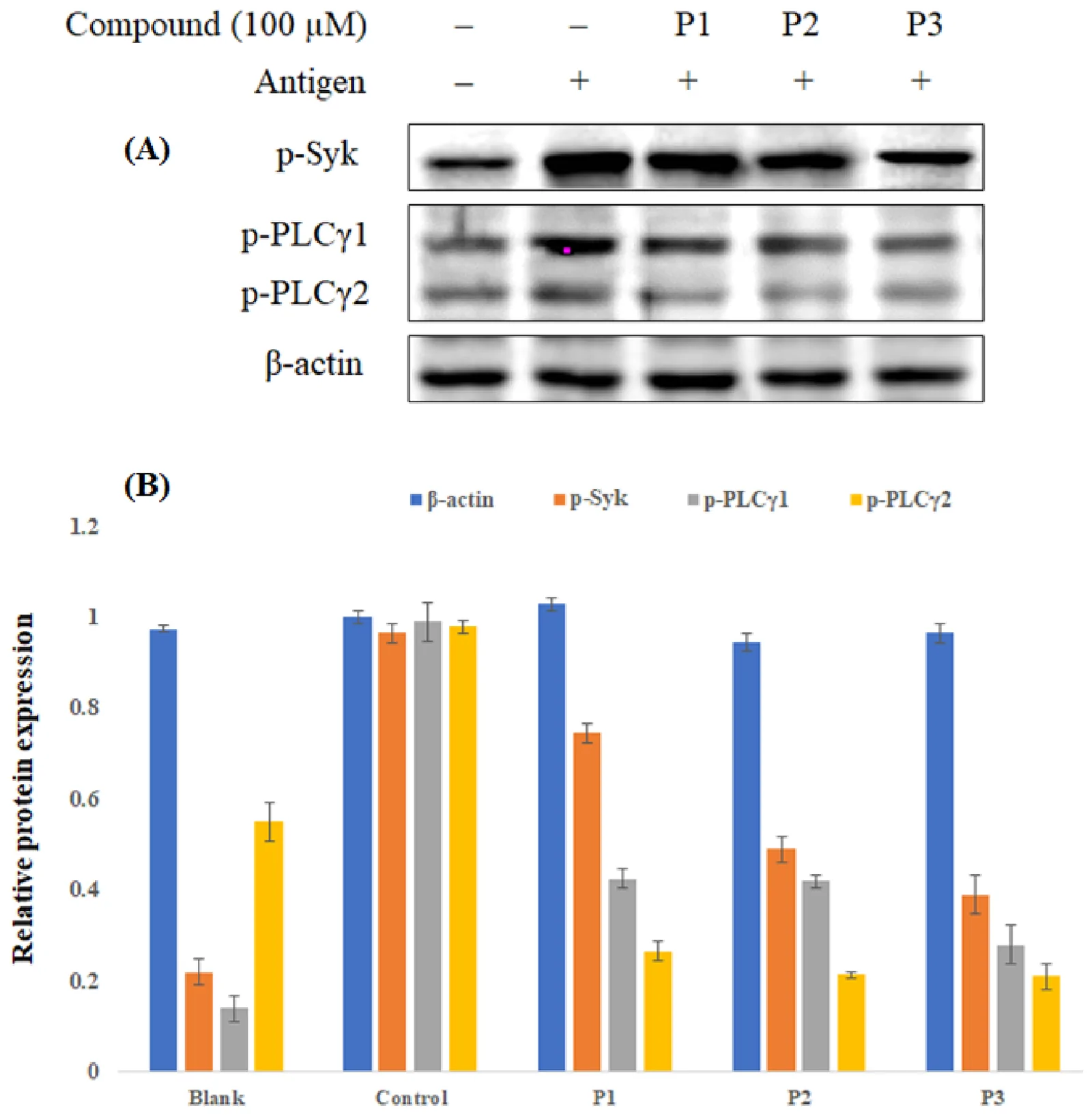
The suppressive effect of the compounds from *P. amarus* on mast cell degranulation-related signaling molecules. RBL-2H3 cells were pre-treated with 100 µM of compounds and sensitized with DNP-specific IgE antibody before stimulation of antigen DNP-BSA. (**A**) The protein expression level was examined by Western blotting assay, and β-actin was used as the internal control. The blank group consisted of unstimulated cells, whereas the control group consisted of DNP-BSA-stimulated cells without compound treatment. (**B**) Quantitative densitometric analysis of p-Syk, p-PLCγ1, and p-PLCγ2 protein expression. Protein band intensities were normalized to β-actin and expressed as relative protein expression. Data are presented as mean ± SD.

Mast cell degranulation is tightly regulated by intracellular signaling pathways, particularly the activation of Syk kinase and downstream PLC-γ 1/2, which mediate calcium mobilization and trigger exocytosis of granule contents [14]. Therefore, inhibition of mast cell degranulation by natural compounds may involve suppression of these signaling cascades. In this context, further investigation of the effects of compounds P**1**, P**2**, and P**3** on allergic signaling pathways is necessary to clarify their mechanisms underlying mediator suppression. The immunoblot analysis demonstrates the effects of compounds P**1**, P**2**, and P**3** (100 μM) on the phosphorylation of key signaling proteins involved in mast cell degranulation. Antigen stimulation markedly increased phosphorylation of Syk, PLCγ1, and PLCγ2 compared with the unstimulated control, confirming efficient induction of the degranulation pathway. Treatment with the test compounds resulted in a noticeable reduction in phosphorylation intensity, as reflected by lighter bands relative to the antigen-only control (Figure 4). Among the compounds, P**2** and P**3** produced the most pronounced inhibition, especially at the level of PLCγ1 and PLCγ2, indicating strong suppression of downstream signaling events critical for calcium mobilization and mediator release. P**1** also exhibited substantial inhibitory effects on both Syk and PLCγ isoforms, though to a slightly lesser extent than P**2** and P**3**. These results suggest that the inhibitory activity of these purified compounds on mast cell degranulation may be mediated, at least in part, through a blockade of the Syk/PLCγ signaling cascade. Such findings are consistent with previous reports that inhibition of Syk or PLCγ phosphorylation effectively stabilizes mast cells and suppresses mast cell degranulation [15,16,17]. The observed suppression of Syk and PLCγ phosphorylation by compounds may result from two possible modes of action. First, phenolic compounds can interact with cell surface receptors (e.g., FcεRI), thereby preventing receptor clustering and subsequent blockage of Syk recruitment [18,19]. Alternatively, these compounds may penetrate the cell membrane and directly interfere with intracellular kinases such as Syk or downstream effectors like PLCγ. Previous studies have shown that flavonoids, including luteolin and kaempferol, inhibit mast cell activation by directly binding and suppressing Syk kinase activity [20], while other polyphenols were reported to modulate both receptor-level and cytoplasmic signaling events [15]. Thus, the inhibitory effects of these compounds could be mediated by a dual mechanism involving both membrane receptor interaction and direct intracellular kinase inhibition. Further mechanistic studies, including kinase binding assays and molecular docking, are warranted to clarify their precise targets.

Overall, compounds P**2** and P**3** exhibited stronger inhibitory effects on mast cell degranulation than P**1**. These differences may be associated with their distinct chemical structures; however, the present data are insufficient to establish a definitive structure–activity relationship or to identify the specific structural features responsible for their activities. Further studies, such as molecular docking, target-binding assays, and evaluation of structurally related analogs, are required to clarify the underlying structure–activity relationships. While the present study demonstrates the inhibitory potential of compounds isolated from *P. amarus*, some limitations should be acknowledged. The current findings are derived from in vitro experiments using the RBL-2H3 mast cell model, which provides a useful platform for investigating IgE-mediated degranulation but does not fully reflect the complexity of allergic responses in vivo. In addition, in vivo efficacy and systemic safety of the identified compounds were not assessed in this study. Future investigations may therefore include validation of these compounds in appropriate animal models of allergy, along with studies on bioavailability and toxicity. Further mechanistic analyses, such as target-binding assays and computational modeling, could also help to clarify their molecular interactions and support the development of these compounds as potential anti-allergic agents.

## 3. Experimental Section

### 3.1. Materials

*P. amarus* was collected in Thu Dau Mot City, Binh Duong province, Vietnam. *P. amarus* was identified by Dr. Dang Le Anh Tuan. A voucher specimen (PHH1004946) was deposited in the Botany Lab, Department of Ecology and Evolutionary Biology, Faculty of Biology and Biotechnology, University of Science—Vietnam National University Ho Chi Minh City. Dulbecco’s Modified Eagle Medium (DMEM) and fetal bovine serum (FBS) were obtained from Gibco (Thermo Fisher Scientific, Waltham, MA, USA). Reagents used throughout the experiments were primarily obtained from Sigma–Aldrich (St. Louis, MO, USA). Cytokine enzyme immunoassay kits were sourced from Invitrogen (Thermo Fisher Scientific, Carlsbad, CA, USA). For Western blotting, specific antibodies were procured from Cell Signaling Technology (Danvers, MA, USA).

### 3.2. The Extraction and Purification

*P. amarus* was shade-dried and subsequently ground into a fine powder. This powdered material was extracted using 70% ethanol with a solid-to-solvent ratio of 1:8 (*w*/*v*) and maintained at 70 °C for 4 h. The crude extract (P-Et) was subsequently subjected to liquid–liquid partitioning using a sequential solvent extraction method with solvents of increasing polarity. After solvent removal under reduced pressure, four sub-fractions were obtained and designated as follows: petroleum ether fraction (P-PE), chloroform fraction (P-C), ethyl acetate fraction (P-EA), n-butanol fraction (P-B), and water fraction (P-W). These fractions were screened for inhibitory activity on histamine release from mast cells. The potential inhibitory fraction was further subjected to flash column chromatography using silica gel (particle size 0.04–0.063 mm) as the stationary phase and a gradient elution system of CHCl_3_–EtOAc (from 7:3 to 0:10, *v*/*v*), followed by methanol wash. As a result, a total of 7 sub-fractions were achieved from this flash column chromatography. Among them, sub-fractions P-EA.2, P-EA.4, and P-EA.7 exhibited potential inhibitory activity and were therefore further subjected to column chromatography on Sephadex LH-20 with a CHCl_3_–MeOH (7:3, *v*/*v*) solvent system. This process respectively yielded three pure compounds, including P**1** (10 mg), P**2** (24 mg), and P**3** (74 mg). The chemical structures of these compounds were elucidated using mass spectrometry (X500R QTOF instrument using ESI in positive ion mode (ESI^+^)) and nuclear magnetic resonance spectroscopy (NMR). Crude fractions/sub-fractions were tested at 100 μg/mL, while purified compounds were tested at 100 μM. This difference reflects the appropriate expression of concentration for chemically undefined mixtures versus structurally identified pure compounds. The concentrations of 100 μg/mL for fractions/sub-fractions and 100 μM for purified compounds were selected as screening concentrations because they allowed clear discrimination of histamine-release inhibitory activity among the tested samples, whereas lower concentrations resulted in less distinct differences between groups.

### 3.3. Cell Culture and Cell Viability

RBL-2H3 cells were maintained at 37 °C in a humidified incubator with 5% CO_2_, using Dulbecco’s Modified Eagle Medium (DMEM) supplemented with 10% heat-inactivated fetal bovine serum (FBS), 2 mM L-glutamine, 10 mM HEPES buffer, 100 units/mL of penicillin G, and 100 µg/mL of streptomycin. Cell viability was assessed using the MTT (3-[4,5-dimethylthiazol-2-yl]-2,5 diphenyl tetrazolium bromide) assay. Briefly, the cells were treated with the extract/compounds for a period of 24 h. Following incubation, the culture medium was discarded and replaced with MTT solution (0.5 mg/mL), which was incubated with the cells for an additional 4 h. After removing the supernatant, DMSO was added to dissolve the formazan crystals formed. The absorbance of each sample was then measured at 540 nm using a microplate reader (Accuris SmartReader 96, Edison, NJ, USA). Cell viability was expressed as a percentage relative to the untreated control group.

### 3.4. Degranulation Assay

Histamine released from cells was measured using a fluorescence spectrophotometric assay [21]. Mast cells were seeded into 24-well plates at a density of 2 × 10^5^ cells/mL. The cells were treated with the extracts/compounds for 6 h prior to overnight sensitization with dinitrophenyl-specific immunoglobulin E (DNP-specific IgE) at a final concentration of 1 µg/mL. Sensitized cells were washed twice with Tyrode buffer (137 mM NaCl; 2.7 mM KCl; 0.4 mM NaH_2_PO_4_; 1 mM MgCl_2_; 12 mM NaHCO_3_; and 1.8 mM CaCl_2_) and subsequently stimulated with dinitrophenyl-bovine serum albumin (DNP-BSA) at a final concentration of 1 µg/mL for 60 min. The supernatant was collected and centrifuged to remove cell death. Then, 40 µL of 0.5 N NaOH and 20 µL of O-phthalaldehyde (OPA, 2.5 mg/mL) were added to 100 µL of the supernatant and incubated for 30 min. The reaction was terminated by adding 10 µL of 3 N HCl. Fluorescence intensity was measured using an excitation wavelength of 365 nm and an emission wavelength of 465 nm. The supernatant from unstimulated cells was used as the blank, while that from DNP-BSA-stimulated cells served as the control. The percentage of histamine release was calculated using the following formula:

Histamine release (%) = [(Fluorescence intensity of test sample − Fluorescence intensity of blank)/(Fluorescence intensity of control − Fluorescence intensity of blank)] × 100.

### 3.5. Measurement of Cytokine Production

RBL-2H3 cells were seeded at a density of 5 × 10^4^ cells/mL and treated with samples for 6 h. Following this treatment, the cells were sensitized overnight using 1 µg/mL of DNP-specific IgE and subsequently challenged with DNP-BSA (1 µg/mL) for another 12 h incubation period. The levels of TNF-α and IL-4 released into the culture medium were quantified according to the manufacturer’s instructions provided by Invitrogen.

### 3.6. Western Blot Analysis

RBL-2H3 cells were treated with the samples for 6 h prior to overnight sensitization with dinitrophenyl-specific immunoglobulin E (DNP-specific IgE) at a final concentration of 1 µg/mL. Sensitized cells were washed twice with Tyrode buffer and subsequently stimulated with dinitrophenyl-bovine serum albumin (DNP-BSA) at a final concentration of 1 µg/mL for 30 min. The cells were collected and lysed using RIPA lysis buffer. Equal amounts of total protein were separated on a 10% SDS-PAGE gel, transferred onto a nitrocellulose membrane, and blocked with BSA. The membrane was then incubated with the appropriate primary antibody for at least 1 h. After washing three times with TBS-T buffer, the membrane was incubated with a horseradish peroxidase (HRP)-conjugated secondary IgG antibody for 1 h. The membrane was then thoroughly washed with TBS-T buffer. Protein bands were visualized using electrochemiluminescence (ECL) detection [22].

### 3.7. Statistical Analysis

Data are presented as mean ± standard deviation (n = 3). Statistical significance was determined using one-way analysis of variance (ANOVA) with SPSS software 25, with a significance level of *p* < 0.05. Graphs and images were generated using Excel software Microsoft 365.

## 4. Conclusions

In conclusion, three purified compounds from *P. amarus* were identified and shown to exhibit inhibitory activity on mast cell degranulation through suppression of histamine release and cytokine production. Among them, ethyl brevifolincarboxylate (P**2**) and 4′,4′′′-di-O-methyl cupressuflavone (P**3**) showed stronger inhibitory effects, which were associated with reduced phosphorylation of Syk and PLCγ. These findings provide preliminary evidence that purified constituents from *P. amarus* may contribute to the anti-allergic activity of this plant and offer a useful basis for further studies on their mechanisms of action and potential biological relevance.

## Figures and Tables

**Table 1:** NMR data of compound P**1** (150/600 MHz, DMSO-*d*_6_).

C	P1 (DMSO, 150/600 MHz)		
	CHn	δ_C_ ppm	δ_H_ ppm (m, J Hz, mH)
1	C_IV_	111.8	
2	C_IV_	141.4	
3	C_IV_	140.7	
4	C_IV_	153.7	
5	CH	107.4	7.61 (s, 1H)
6	C_IV_	113.3	
7	C_IV_	158.4	
1′	C_IV_	111.1	
2′	C_IV_	140.9	
3′	C_IV_	140.1	
4′	C_IV_	152.5	
5′	CH	111.6	7.51 (s, 1H)
6′	C_IV_	112.4	
7′	C_IV_	158.2	
3-OMe	CH_3_	61.2	4.04 (s, 3H)
4-OMe	CH_3_	56.6	4.00 (s, 3H)
3′-OMe	CH_3_	60.9	4.05 (s, 3H)

**Table 2:** NMR data of compound P**2** (150/600 MHz, DMSO-*d*_6_).

C	P2 (DMSO, 150/600 MHz)	
	δ_C_ ppm	δ_H_ ppm (m, J Hz, mH)
2	145.7	
3	138.4	
4	114.9	
5	112.9	
6	108.0	7.30 (s, 1H)
7	149.6	
8	140.2	
9	143.6	
10	160.1	
11	40.7	4.40 (dd, J = 7.7, 2.0 Hz, 1H)
12	37.0	2.98 (dd, J = 18.6, 7.7 Hz, 1H) 2.44 (dd, J = 18.6, 2.0 Hz, 1H)
13	192.9	
1′	171.9	
2′	60.5	4.09 (q, J = 7.1 Hz, 2H)
3′	13.9	1.17 (t, J = 7.1 Hz, 3H)

**Table 3:** NMR data of compound P**3** (150/600 MHz, DMSO-*d*_6_).

C	P3 (DMSO, 150/600 MHz)	
	δ_C_ ppm	δ_H_ ppm (m, J Hz, mH)
1		
2	163.2	
3	103.3	6.88 (s, 1H)
4	182.0	
5	159.9	
6	94.0	6.52 (s, 1H)
7	160.7	
8	114.0	
9	156.8	
10	103.1	
1′	122.6	
2′	114.5	7.11 (d, J = 9.0 Hz, 1H)
3′	128.3	8.04 (d, J = 8.9 Hz, 1H)
4′	162.3	
5′	128.3	8.04 (d, J = 8.9 Hz, 1H)
6′	114.5	7.11 (d, J = 9.0 Hz, 1H)
5-OH		12.69 (s, 1H)
6-OH		13.85 (s, 1H)
4′-OCH3	55.5	3.86 (s, 3H)

## Data Availability

The original contributions presented in this study are included in the article/Supplementary Materials. Further inquiries can be directed to the corresponding author.

## Supplementary Materials

The following supporting information can be downloaded at: https://www.mdpi.com/article/10.3390/molecules31162835/s1.
