# Supplementary material for: Purified Compounds from Phyllanthus amarus Schum. & Thonn. Inhibit Mast Cell Degranulation via Modulation of Syk/PLCγ Signaling
Source: Molecules. 2026 Aug 14;31(16):2835. doi: 10.3390/molecules31162835 (PMC13515741; doi:10.3390/molecules31162835)
Supplement: Supplementary file 1 [file molecules-31-02835-s001.zip › molecules-4431336-supplementary.pdf]

Purified compounds from *Phyllanthus amarus* Schum. & Thonn. inhibit mast cell degranulation via modulation of Syk/PLCγ signaling

Supplementary Materials

Figure S1

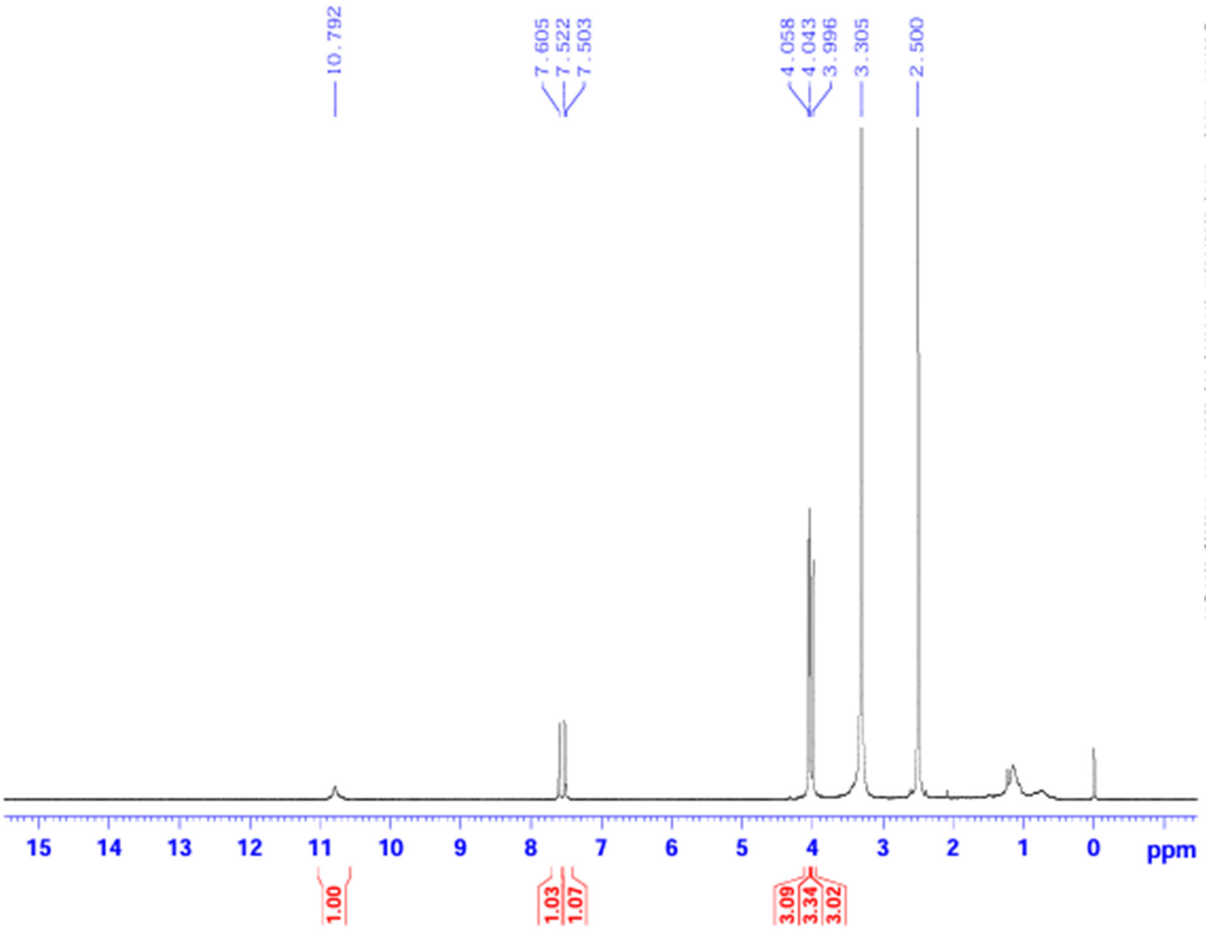

Figure S1.1. ^{1}H NMR spectrum of 3,3',4-tri-O-methylellagic acid (P**1**)

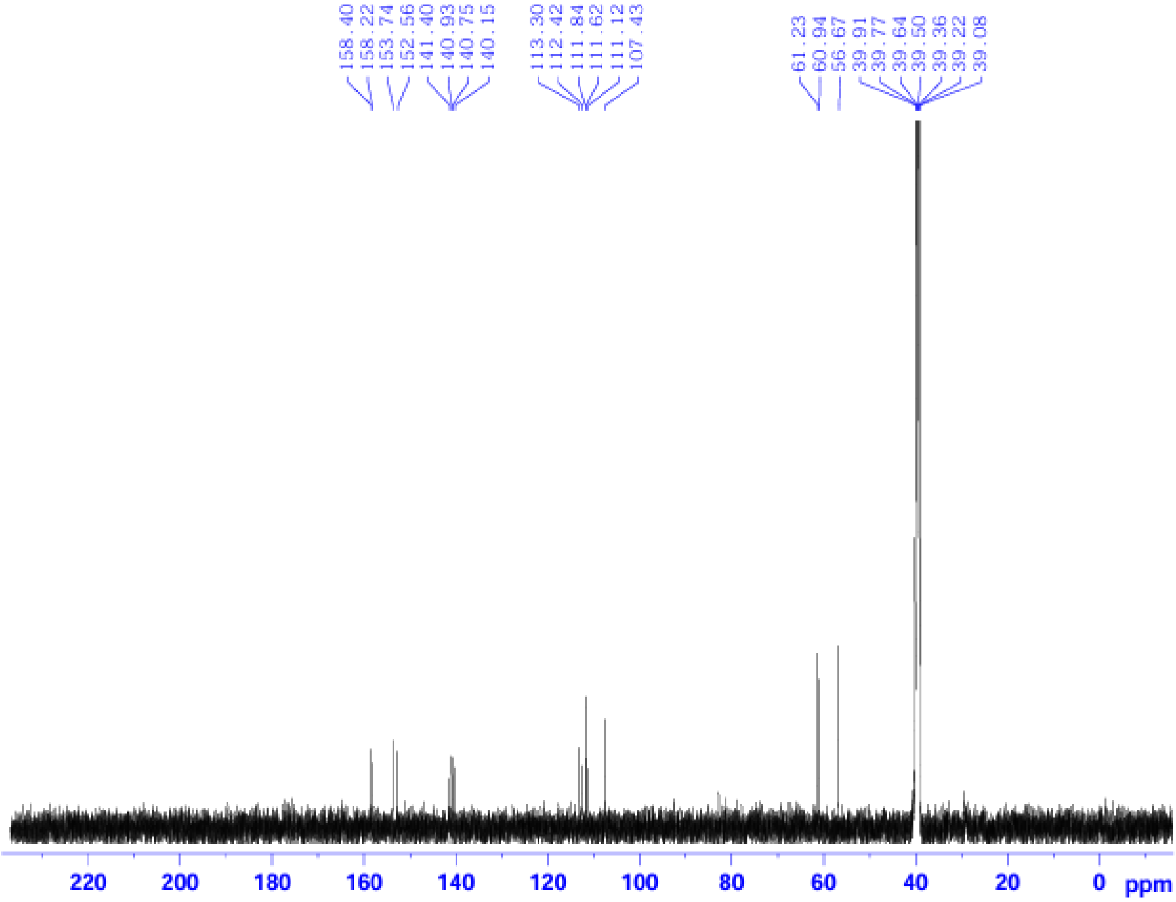

Figure S1.2. ^{13}C NMR spectrum of 3,3',4-tri-O-methylellagic acid (P**1**)

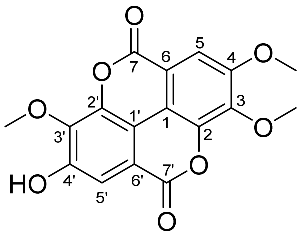

Figure S1.3. The chemical structures of 3,3',4-tri-O-methylellagic acid (P1)

Figure S2

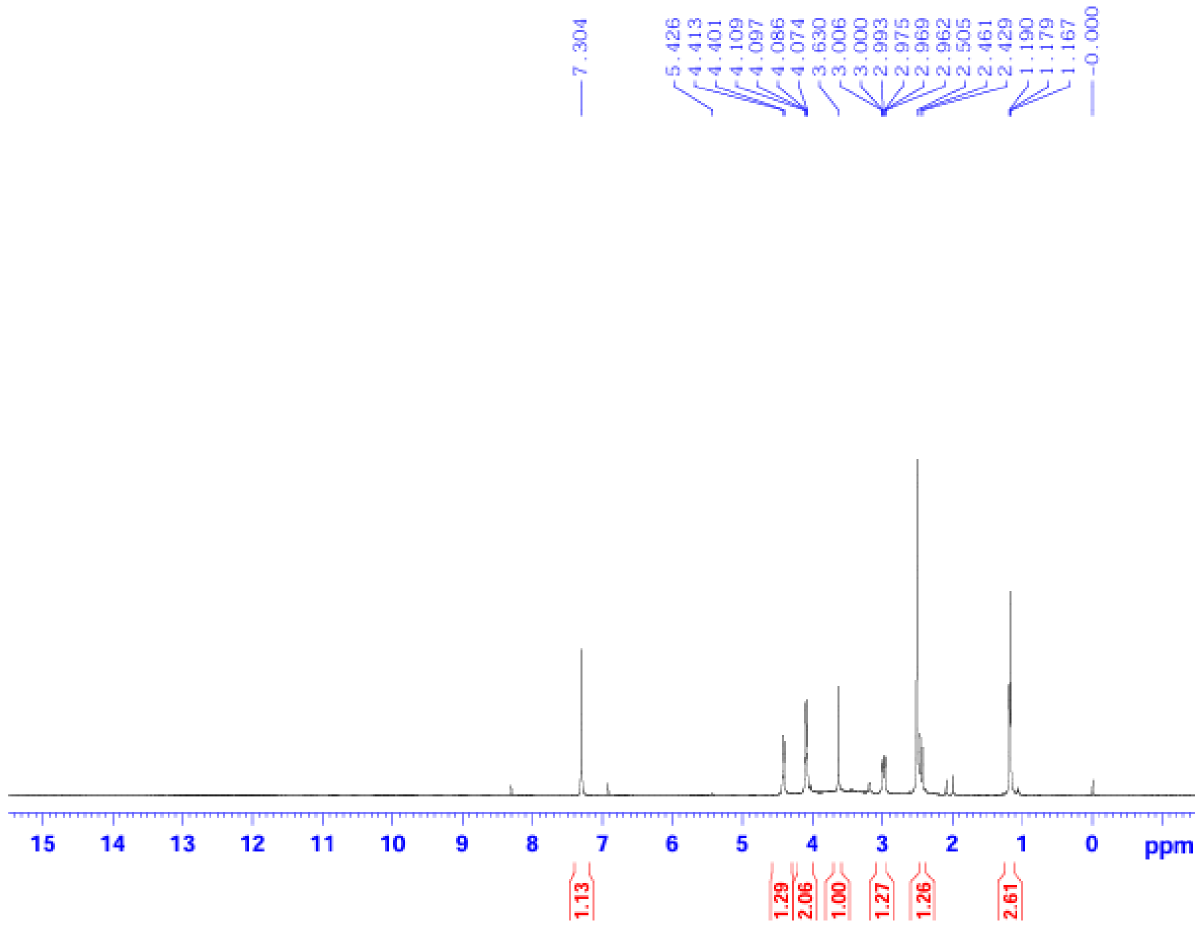

Figure S2.1. ^{1}H NMR spectrum of ethyl brevifolincarboxylate (P**2**)

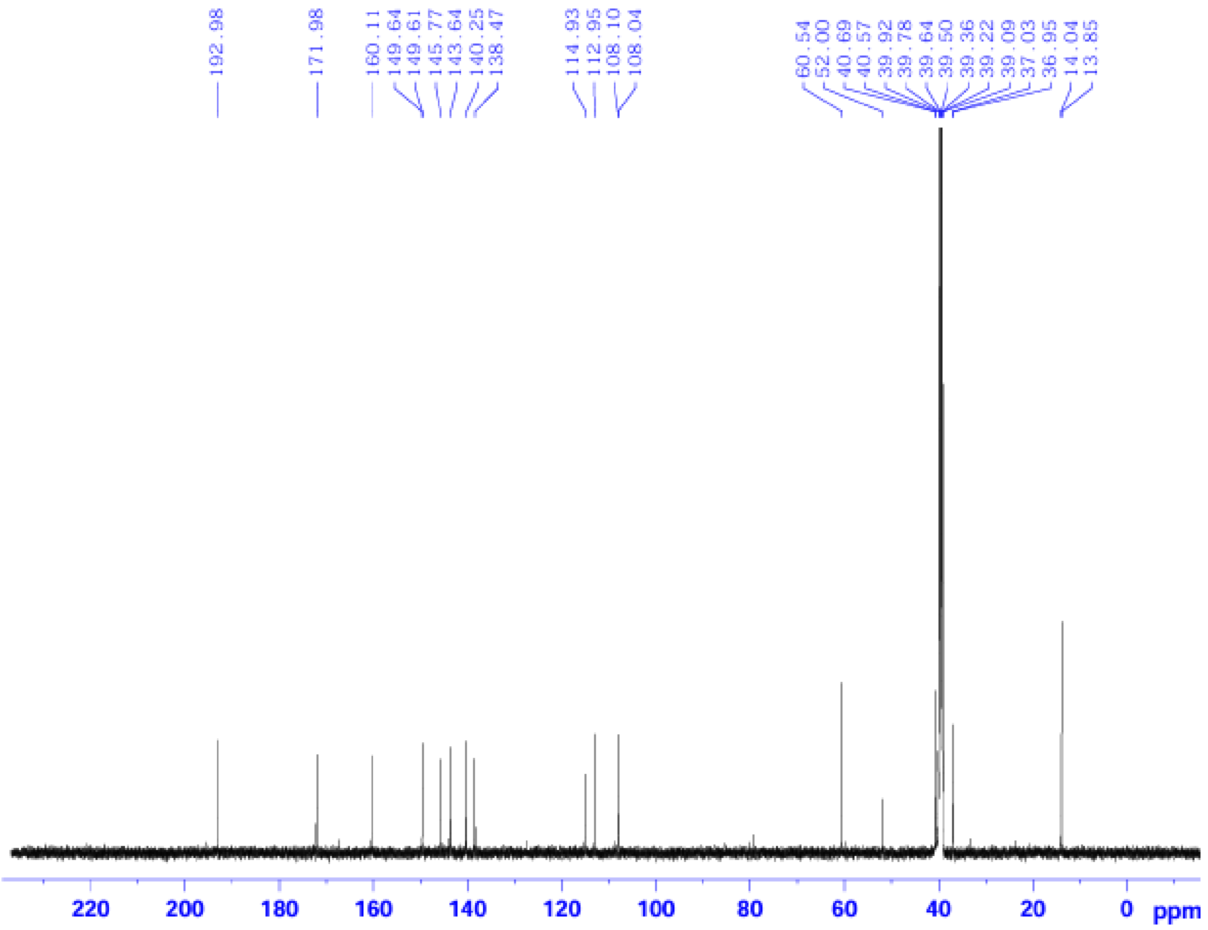

Figure S2.2. ^{13}C NMR spectrum of ethyl brevifolincarboxylate (P**2**)

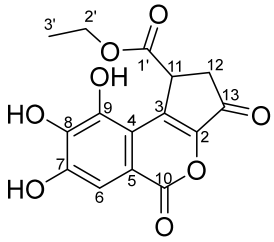

Figure S2.3. The chemical structures of ethyl brevifolincarboxylate (P**2**)

Figure S3

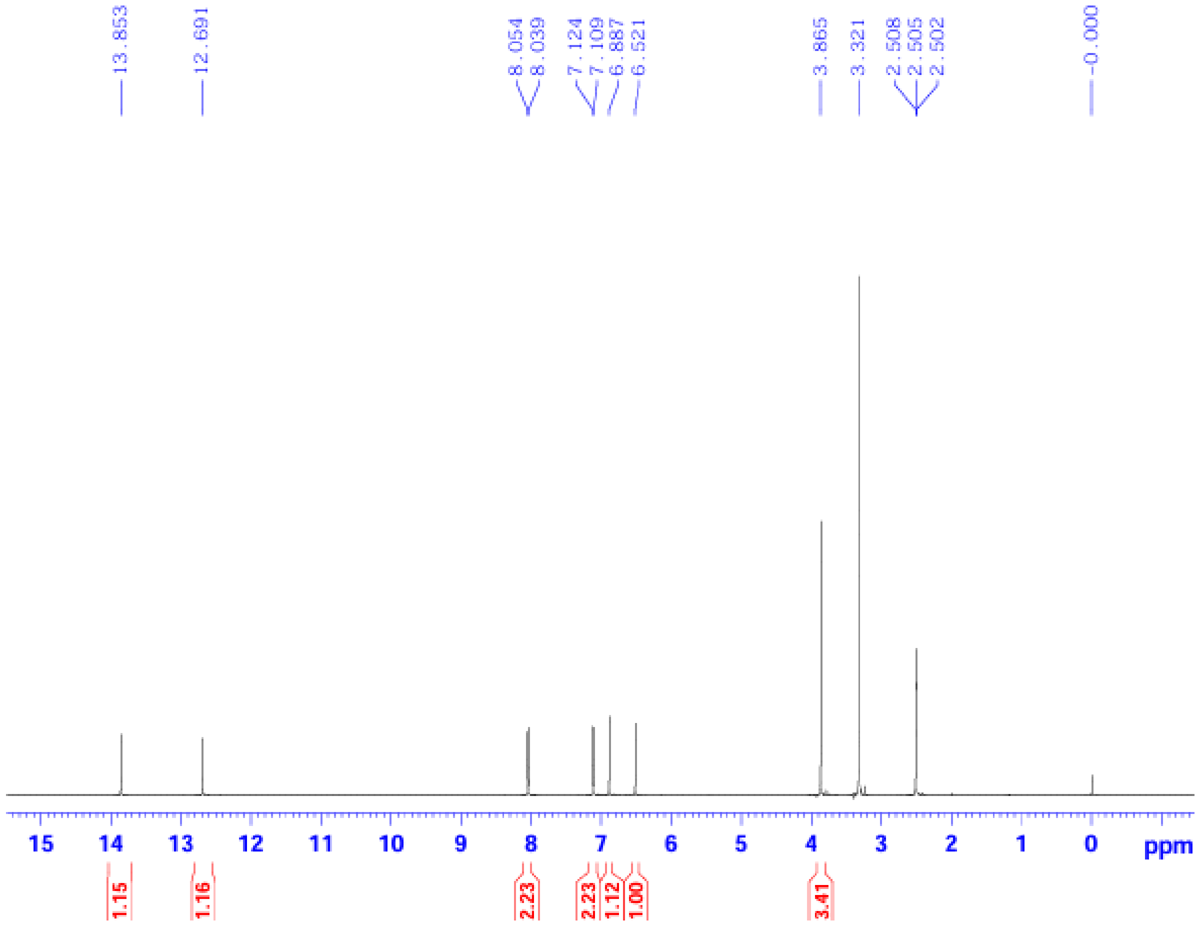

Figure S3.1. ^{1}H NMR spectrum of 4′,4‴-di-O-methyl cupressuflavone (P**3**)

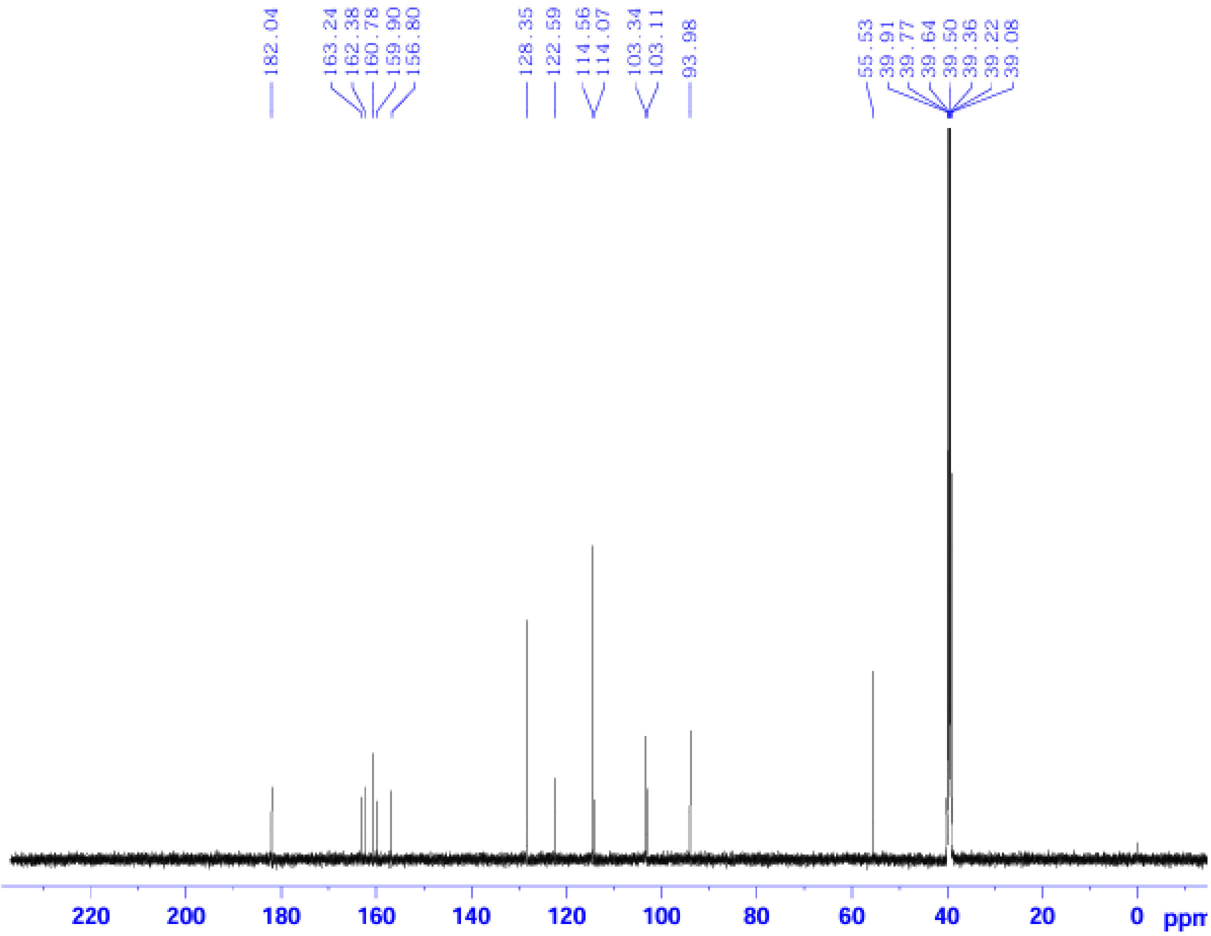

Figure S3.2. ^{13}C NMR spectrum of 4′,4‴-di-O-methyl cupressuflavone (P**3**)

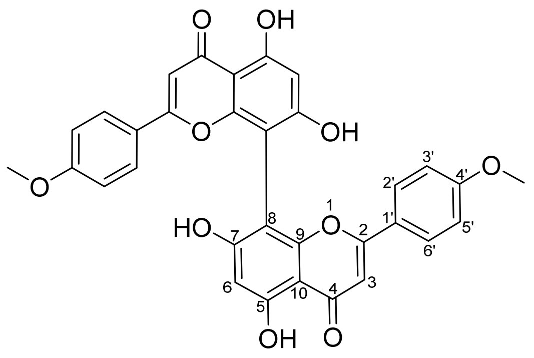

Figure S3.3. The chemical structures of 4′,4′′′-di-O-methyl cupressuflavone (P**3**)
